# Transgenic Cotton Expressing ds*AgCYP6CY3* Significantly Delays the Growth and Development of *Aphis gossypii* by Inhibiting Its Glycolysis and TCA Cycle

**DOI:** 10.3390/ijms26010264

**Published:** 2024-12-31

**Authors:** Wenting Kong, Tingting Li, Yuan Li, Lianjun Zhang, Jingang Xie, Xiaoning Liu

**Affiliations:** Xinjiang Key Laboratory of Biological Resources and Genetic Engineering, College of Life Science and Technology, Xinjiang University, Urumqi 830017, China; 15562268925@163.com (W.K.); ltt1554970302@163.com (T.L.); liyuan010517@163.com (Y.L.); 107556521140@stu.xju.edu.cn (L.Z.); 107556522145@stu.xju.edu.cn (J.X.)

**Keywords:** *Aphis gossypii*, transcriptome, metabolome, glycometabolism, transgenic cotton

## Abstract

In our previous research, we found that *CYP6CY3* not only participates in the detoxification metabolism of neonicotinoid insecticides in cotton aphid but also affects their growth and development. However, how does transgenic cotton expressing ds*AgCYP6CY3* affect the growth and development of cotton aphid? In this study, we combined transcriptome and metabolome to analyze how to inhibit the growth and development of cotton aphid treated with transgenic cotton expressing ds*AgCYP6CY3-P1* (TG cotton). The results suggested that a total of 509 differentially expressed genes (DEGs) were identified based on the DESeq method, and a total of 431 differential metabolites (DAMs) were discovered using UPLC-MS in the metabolic analysis. Additionally, multiple DEGs and DAMs of glycolytic and The tricarboxylic acid (TCA) cycle pathways were significantly down-regulated. Pyruvate carboxylase (PC), citrate synthase (CS), malate dehydrogenase (MDH) enzyme activities and pyruvate content were reduced in cotton aphid treated with TG cotton. In addition, TG cotton could significantly decrease the total sugar content from the body and honeydew in cotton aphid. The above results indicated that TG cotton inhibited glycolysis and the TCA cycle, and this inhibition is consistent with previous studies showing that cotton aphid fed on TG cotton showed significantly reduced body length and weight as well as delayed molting. These findings provide a new strategy for reducing the transmission of viruses by cotton aphid honeydew, preventing fungal growth, mitigating impacts on normal photosynthesis and improving cotton quality.

## 1. Introduction

Cotton (*Gossypium hirsutum* L.) is a crucial cash crop in China, serving not only as a strategic material critical to the national economy but also as one of the major agricultural products worldwide [1]. With the large-scale cultivation of cotton, the ecological function of the concentrated cotton growing areas have become very fragile. Cotton was extremely vulnerable to the threat of pests and diseases. In recent decades, the cultivation of Bt cotton in large areas has effectively controlled the damage caused by lepidopteran insects such as *Helicoverpa armigera* while affecting the population dynamics and community structure of nontarget pests [2,3]. For instance, the population of *Aphis gossypii* Glover (Homoptera: Aphididae) has tended to increase, and its small size, high fecundity capacity and strong adaptability make it one of the main pests that need to be controlled in cotton fields [4,5]. The cotton aphid is a cosmopolitan and polyphagous pest with a wide variety of hosts, such as *G. hirsutum*, *Cucumis sativus*, *Citrus reticulata* and many other crops globally [6,7], and it mainly gathers on the back of the young leaves of cotton to suck the sap. Honeydew (grease) is the excreta of cotton aphid, which affects the normal photosynthesis of the plants, contaminating the cotton fibers, inducing mildew parasitism and carrying a wide range of plant viruses as vectors [8,9,10]. Honeydew is a food source for other insects and consists mainly of sucrose, glucose and fructose [11,12]. Consequently, it is urgent to explore new, eco-friendly pest control strategies to reduce the contamination caused by cotton aphid honeydew to enhance cotton quality.

Cytochrome P450s (CYP450s) play an important role in the growth, development and glycolipid metabolism in many insects [13,14,15,16]. For instance, silencing the *CYP314A1* gene from the 3rd instar larvae of *Bactrocera minax* has resulted in a significant reduction in trehalose (28.29% decrease) and glycogen (14.55% decrease), larval mortality and abnormal pupation compared to dsGFP-treated controls [17]. Similarly, the silencing of the *TcCYP6K1* and *TcCYP9F2* genes has hindered trehalose metabolism in *T. castaneum*, led to an insufficiency of trehalase enzymes for trehalose breakdown and ultimately caused insect mortality due to the inability to maintain normal physiological activities [18]. Their results clarified that the inhibition of the glycometabolism by interfering with cytochrome P450 in insects led to developmental arrest and the disruption of chitin synthesis. A similar finding was made in cotton aphid; silencing *CYP6CY19* significantly increased larval and adult mortality and reduced total fecundity [19]. Furthermore, the mortality rate of *A. gossypii* significantly increased when Ace-R strains treated with ds*CYP6CY14*, ds*CYP6DC1* and ds*CYP6CZ1*, respectively [20]. Our previous results showed that the nanomaterial-mediated and plant-mediated silencing of *CYP6CY3* has increased the sensitivity of cotton aphid to neonicotinoid insecticides, reduced their population density and delayed their growth and development [21,22]. These studies suggested that silencing *CYP6CY3* of the cotton aphid affected growth and development, but the specific reasons for this impact remain unclear.

In this study, we analyzed the effect of expressing ds*AgCYP6CY3-P1* cotton on the growth and development of cotton aphid based on transcriptome and metabolome. We found that the DEGs and DAMs co-enriched in glycolysis and the TCA cycle. In addition, the expression levels of genes involved in glycolysis and the TCA cycle pathway were analyzed by the reverse transcription quantitative PCR (RT-qPCR) method. PC, CS and the MDH enzyme activities and pyruvate content were measured. The total sugar content from the body and the honeydew in cotton aphid were also examined. These results will lay the foundation for exploring the mechanisms between developmental retardation and glycolysis and the TCA cycle of the cotton aphid treated with expressing ds*AgCYP6CY3-P1* cotton.

## 2. Results

### 2.1. Effect of TG Cotton on Transcriptome of Cotton Aphid

**Figure 1:**
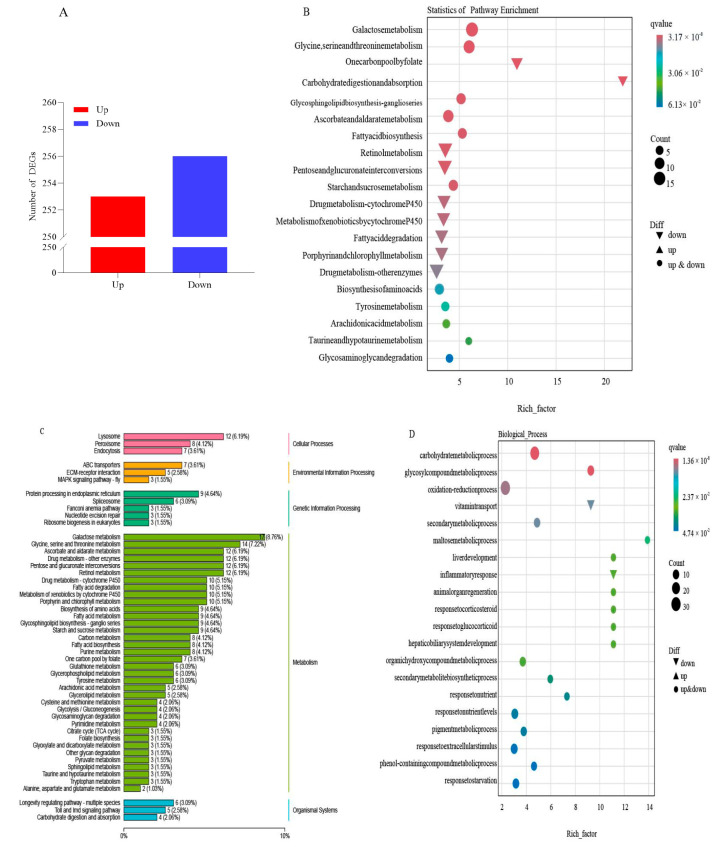

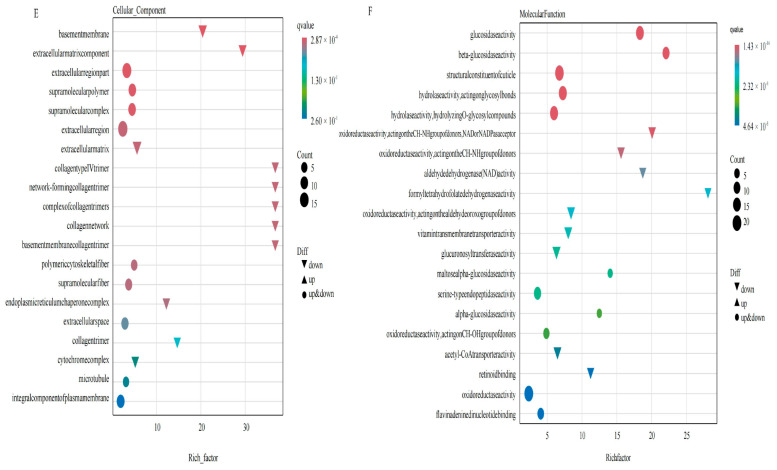
Overview of transcriptome of cotton aphid fed on TG cotton. (**A**) Up- and down-regulated DEGs. (**B**) KEGG pathway enrichment analysis of DEGs. Circle sizes indicate total number of enriched genes. X-axis shows enrichment factor. (**C**) KEGG classification enrichment analysis of DEGs. (**D**–**F**) Functional categories of DEGs in molecular functions (MFs), biological processes (BPs) and cellular components (CCs). Circle sizes indicate total number of enriched genes. X-axis shows enrichment factor.

The transcriptomic analysis revealed a total of 509 differentially expressed genes in cotton aphid fed on NT and TG cotton, of which 253 DEGs were up-regulated and 256 DEGs were down-regulated (Figure 1A) (Supplementary Table S1). To better understand the functions of these DEGs, a GO enrichment and KEGG analysis were performed. The KEGG analysis indicated that the DEGs were enriched in 96 pathways. The TOP 20 enrichment pathway in the DEGs was related to galactose metabolism, glycine, serine and threonine metabolism, carbohydrate digestion and absorption and fatty acid biosynthesis drug metabolism-cytochrome P450 (Figure 1B). In particular, the expression of 80 glycometabolism genes were significantly altered (Figure 1C). The GO analysis revealed that the DEGs were categorized into three main groups, namely biological processes (BPs), cellular components (CCs) and molecular functions (MFs). In the BP category, the Top3 enriched pathways were the carbohydrate metabolic process, the glycosyl compound metabolic process and the oxidation–reduction process. Glucosidase activity, hydrolase activity and oxidoreductase activity were significantly enriched in the CC category. Additionally, in the MF category, the enriched pathways were the basement membrane extracellular matrix component and the collagen trimer (Figure 1D–F). Taken together, the results showed that multiple DEGs were enriched in the glycometabolism pathway, and they mainly participated in the glycometabolism process by exerting glycosidase activity, hydrolase activity and oxidoreductase activity.

### 2.2. Effect of TG Cotton on Metabolome of the Cotton Aphid

**Figure 2:**
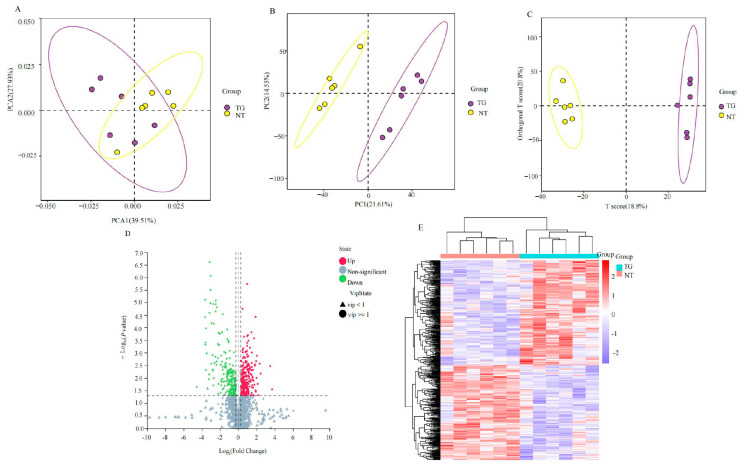
Overview of metabolome of cotton aphid fed on TG cotton. (**A**–**C**) PCA, PLS-DA and OPLS-DA score plots of metabolomics data obtained by LC-MS/MS analysis of cotton aphid fed on TG cotton and NT cotton. (**D**) Volcano map of DAMs in cotton aphid fed on TG cotton and NT cotton. Horizontal coordinates were log2-transformed fold change; vertical coordinates were -log10-transformed *p*-value. Down-regulated significantly different metabolites shown in blue, up-regulated significantly different metabolites shown in red, and non-significant metabolites grayed out. (**E**) Heatmap of main differential metabolites on cotton aphid treated with NT cotton and TG cotton. Metabolites content highlighted in red (up-regulated) and violet (down-regulated).

We compared the metabolomic differences between cotton aphid fed on TG cotton and NT cotton at 48 h based on a multivariate statistical analysis, including the unsupervised PCA and supervised PLS-DA and OPLS-DA methods. In the present study, as shown in Figure 2, the PCA scoring plot initially showed differences in major metabolites between cotton aphid fed on TG cotton and NT cotton (Figure 2A). The PLS-DA score plots (Figure 2B) show significant differences between the two treatments. In addition, the OPLS-DA score plots (Figure 2C) further showed a significant separation between the two treatments, where the model performance parameters, R2Y (cum) and Q2 (cum), were 0.994 and 0.666, respectively, indicating the high discriminatory and predictive ability of the model. A total of 431 differentially expressed metabolites (DAMs) were identified as significantly expressed by cotton aphid fed on TG cotton at 48 h, of which 227 were up-regulated and 204 were down-regulated (Figure 2D). The heat map of the differential metabolite clustering (Figure 2E) clearly shows that these DAMs include threonine, histidine, pyruvate, N-acyl-D-galactosamine, mannitol, citrate, uridine 5′-monophosphate and aflatoxin B1. These results indicated that a variety of differential metabolites were enriched in some metabolic pathways such as amino acid metabolism, sugar metabolism and the cofactor synthesis of cotton aphid feeding on TG cotton.

### 2.3. DEGs and DAMs Co-Enriched in Glycolysis and TCA Cycle Based on Transcriptomic and Metabolomic Analyses

**Figure 3:**
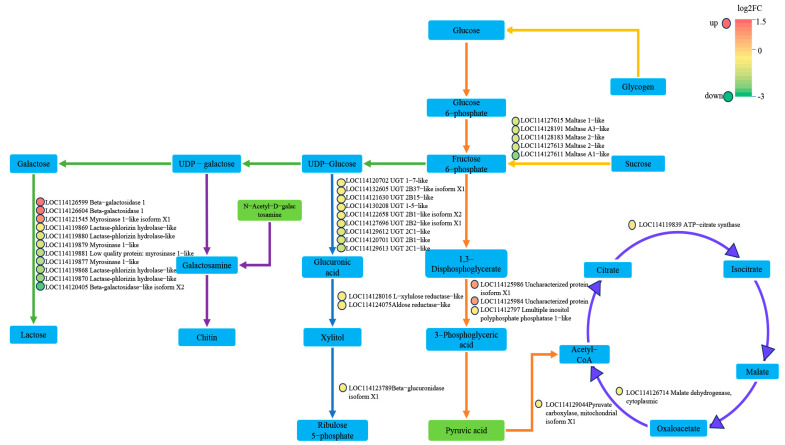
The DAMs and related DEGs involved in glycometabolism. The rectangles represent the metabolites. The colors of the rectangles indicate the significances presented on a color scale. Green indicates down significance. The circles indicate the DEGs. The colors indicate the significances shown on a color scale.

Combined transcriptome and metabolome analyses mapped the KEGG pathway and revealed that the DEGs and DAMs were mainly enriched in multiple glycometabolic pathways, including the galactose metabolism pathway, pentose phosphate pathway, glycolysis and the TCA cycle pathway. These DEGs and DAMs were significantly down-regulated, with 11 DEGs enriched in the galactose metabolism pathway and 12 DEGs enriched in the pentose phosphate pathway. The differential metabolite N-acetylgalactosamine involved in chitin synthesis. The pyruvate and 6 DEGs co-enriched in the glycolysis and TCA cycle pathways, and the pyruvate served as a key link between the two pathways (Figure 3). These results showed that the DEGs and DAMs involved in the glycometabolic pathways were significantly down-regulated in cotton aphid fed on TG cotton, and they were also co-enriched in glycolysis and the TCA cycle by combined transcriptome and metabolome analyses.

### 2.4. Effects on Glycolysis and TCA Cycle of Cotton Aphid Fed on TG Cotton

**Figure 4:**
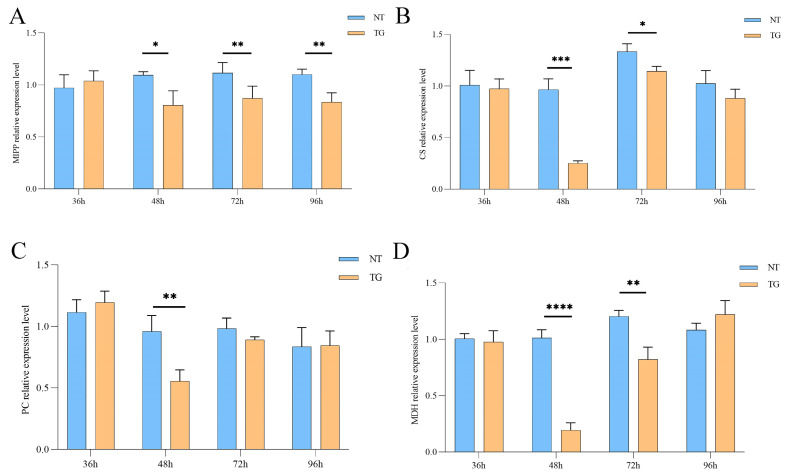
Effects of TG cotton on glycolysis and TCA cycle in cotton aphid. (**A**–**D**) Relative expression levels of multiple inositol polyphosphate phosphatase (*MIPP*), citrate synthase (*CS*), pyruvate carboxylase (*PC*) and malate dehydrogenase (*MDH*) genes in cotton aphid were determined by RT-qPCR at 24 h, 48 h, 72 h and 96 h after being fed on NT and TG cotton. Data are presented as mean ± SD of three independent biological replications. Significant differences between treatment groups and controls were analyzed by Student’s *t*-test, * *p* ≤ 0.05; ** *p* ≤ 0.01; *** *p* ≤ 0.001; **** *p* ≤ 0.0001.

**Figure 5:**
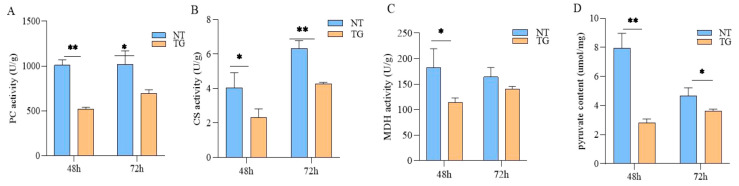
Effects of TG cotton on glycolysis and TCA cycle in cotton aphid. (**A**–**D**) PC, CS, MDH activities and pyruvate content of cotton aphid were measured at 48 h and 72 h treated with NT cotton and TG cotton. Data are presented as mean ± SD of three independent biological replications. Significant differences between treatment groups and controls were analyzed by Student’s *t*-test, * *p* ≤ 0.05; ** *p* ≤ 0.01.

Based on the combined omics techniques analysis, we compared the relative expression levels of DEGs and the pyruvate content in the glycolytic and TCA cycle pathways. Firstly, the relative expression levels of multiple inositol polyphosphate phosphatase (*MIPP*), pyruvate carboxylase (*PC*), citrate synthase (*CS*) and malate dehydrogenase (*MDH*) in cotton aphid fed on TG cotton after 36 h, 48 h, 72 h and 96 h were detected by a qPCR analysis. The results revealed that the relative expression levels of *MIPP*, *PC*, *CS* and *MDH* were all significantly down-regulated (Figure 4A–D). In addition, at the metabolite level, we examined the enzymatic activities of *PC*, *CS* and *MDH* after 48 h and 72 h treated with TG cotton, which were also reduced by 1.94-fold, 1.6-fold and 1.73-fold at 48 h, respectively, compared with the control group. The activities of pyruvate carboxylase and citrate synthase were significantly decreased after the cotton aphid fed on the TG cotton for 72 h (Figure 5A–D), and the content of pyruvate, an intermediate product of the glycometabolism, was much lower than that of the control group. Taken together, the relative expression levels of the *MIPP*, *PC*, *CS* and *MDH* genes were significantly decreased after the cotton aphid fed on TG cotton for 48 h, and both the enzyme activities of PC, CS and MDH and the pyruvate content were also reduced. Together, these results indicated that TG cotton alters both the transcriptomic and metabolomic profiles of cotton aphid, particularly affecting the glycolysis and TCA cycle processes.

### 2.5. Change Profile of Total Sugar Content and Honeydew of Cotton Aphid Treated with TG Cotton

**Figure 6:**
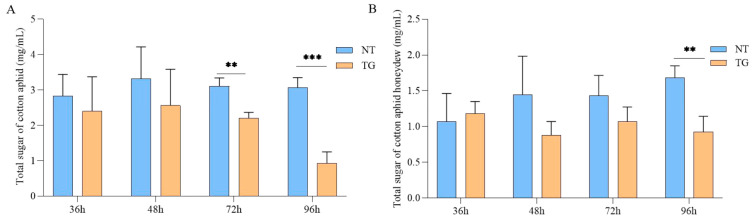
Total sugar content and honeydew of cotton aphid treated with TG cotton. (**A**) Total sugar levels of cotton aphid after feeding on NT and TG cotton for 36 h, 48 h, 72 h and 96 h. (**B**) Total sugar levels of cotton aphid and their honeydew after feeding on NT and TG cotton for 36 h, 48 h, 72 h and 96 h. Data are presented as mean ± SD of three independent biological replications. Significant differences between treatment groups and controls were analyzed by Student’s *t*-test, ** *p* ≤ 0.01; *** *p* ≤ 0.001.

To further evaluate the effects of TG cotton on the glycometabolism in cotton aphid, we determined the total sugar content from the body of cotton aphid and their honeydew. The results indicated that the total sugar content was significantly reduced in cotton aphid treated with TG cotton for 72 h and 96 h, showing a 70% reduction at 96 h (Figure 6A). Honeydew, a final metabolite of the cotton aphid, is highly detrimental to cotton. We measured the total sugar content in the honeydew of cotton aphid following the TG cotton treatment, which showed that the total sugar content in the honeydew was markedly decreased at 96 h, being only 0.55-fold that of the control (Figure 6B). These results indicated that TG cotton could significantly decrease the total sugar content from the body and honeydew in cotton aphid.

## 3. Discussion

Cytochrome P450s play an important role in the growth, development and glycometabolism of many insects [23,24,25]. We observed that silencing *CYP6CY3* from cotton aphid could delay their growth and development and reduce their population density, and the reasons for this phenomenon are still unclear [21,22].

Insect growth and development is a complex physiological process [26]. In recent years, omics techniques have provided new perspectives for understanding these complex physiological responses of individuals [27]. Transcriptome analysis has proven to be an effective method for the large-scale screening of genes associated with specific functions, and the metabolome can provide dynamic changes in all metabolites within an organism [28,29,30]. A combined transcriptome and metabolome analysis allows for more accurate identification of functionally relevant genes, metabolites and enriched pathways. In this study, our results revealed that both DEGs and DAMs were co-enriched in glycolysis and the TCA cycle (Figure 3), and multiple DEGs were significantly down-regulated, including multiple inositol polyphosphate phosphatase, pyruvate carboxylase, citrate synthase and malate dehydrogenase. The above results were tested by RT-qPCR (Figure 4A–D). At the metabolite level, the pyruvate levels were significantly lower when the cotton aphid fed on the TG cotton at 48 h and 72 h. The enzymatic activities of PC, CS and MDH, which were regulated by DEGs, were also significantly reduced. The total sugar levels and final metabolite honeydew levels were significantly reduced in the cotton aphid fed on TG cotton at 96 h (Figure 5A–D). Our previous research illustrated that aphids treated with TG cotton had a prolonged development period of 0.5 days for third-instar nymphs, a significantly reduced cumulative reproductive rate and a significant down-regulation of morphological parameters such as body weight and body length. Therefore, TG cotton delayed the growth and development of cotton aphid by inhibiting glycolysis and the TCA cycle [21,22].

Pyruvate, as a key point connecting the glycolysis and TCA cycle pathways, plays an important role in the growth and development of insects [31]. Pyruvate regulates insect growth and development by multiple pathways. The balance of the pyruvate metabolism in insects is regulated by endogenous hormones (such as hormones and insulin), which affect the growth rate, developmental cycle and reproductive capacity [32,33]. Additionally, pyruvate can regulate insects’ growth and development in non-energy metabolic pathways, such as by participating in fatty acid and amino acid metabolism [34,35,36]. Furthermore, pyruvate enters the mitochondria to participate in the TCA cycle, providing energy for insect growth and development [37]. Similar results were obtained in our study, where the pyruvate levels were reduced and the TCA cycle was inhibited in the cotton aphid treated with TG cotton. Thus, glycolysis and the TCA cycle played a crucial role in providing energy for the insects’ growth and development [38,39,40,41]. Metabolites in the glycolytic and TCA cycle pathways were significantly reduced in Cd-treated larvae compared to controls, and the mRNA expression levels of key regulatory genes (e.g., phosphofructokinase-1, hexokinase-1, citrate synthase) were also lower. In addition, Cd treatment was associated with significant reductions in the larval basal growth data, growth index, body mass index and standardized growth index [42]. Similarly, The glycometabolism and chitin synthesis of *Spodoptera litura* treated with validamycin were inhibited, leading to significant reductions in growth, development and pupation rates [43]. The inhibition of glycolytic and respiratory chain enzymes by 3-BrPA in *Rhipicephalus microplus* also disrupted embryonic development [44]. These results further clarified that the inhibition of glycolysis and the TCA cycle retard insect growth and development.

Glycolysis and the TCA cycle play a vital role in living organisms, providing energy production for cell growth and substrates for anabolism during growth. In our previous study, the bioassay indicated that the growth retardation of cotton aphid was probably caused by anabolism obstruction, which was due to the shortage of ATP caused by TG cotton-induced glycolysis and TCA cycle inhibition [45,46]. TG cotton-induced glycolysis and TCA cycle inhibition reduced intracellular ATP levels. Under low ATP production, AMPK (adenosine 5′-monophosphate (AMP)-activated protein kinase) is activated at high levels of AMP, which leads to the inhibition of mTOR; downstream regulators regulate cell growth and metabolism, resulting in the enhancement of autophagy flux. AMPK is vital for promoting protein synthesis and cell growth [47,48,49]. Thus, it might be a crucial factor that TG cotton caused cotton aphid growth arrest. On the other hand, previous studies have demonstrated that glycolysis and the TCA cycle were related closely to regulations of blood sugar homeostasis and glycogen synthesis as well as insulin secretion and lipogenesis [50,51,52,53]. In other words, the in vivo levels of sugar in cotton aphid were determined by the glycolytic and TCA cycle flux in a way, and a significant reduction in the total sugar content and honeydew of cotton aphid treated with TG cotton resulted. This reduction was probably due to the decrease in carbohydrate intake caused by the TG cotton-induced antifeedant activity. They suggested that antifeedant activity was regulated by a complex neuroendocrine network, which was influenced by energy metabolism. The RNAi knockdown by the injection of dsNPY into *Mythimna separata* significantly inhibited food uptake and body weight, delayed developmental duration and caused an increase in trehalose and decrease in glycogen and total lipids compared to dsGFP [54]. The RNAi-mediated knockdown of sNPF and its receptor coordination led to a feeding reduction, a glycogen and total lipid content decrease, and a growth and development delay in *M. separata* [55]. In conclusion, TG cotton retarded growth and development by inhibiting glycolysis and the TCA cycle of cotton aphid. This may occur through two molecular mechanisms. TG cotton-induced glycolysis and TCA cycle inhibition reduced the intracellular ATP levels, which caused AMPK activation and simultaneously inhibited anabolic growth signaling. In addition, TG cotton-induced antifeedant activity attributed to the carbohydrate intake.

In summary, the PC, CS and MDH enzyme activities and pyruvate content were significantly decreased in cotton aphid treated with TG cotton. Lower total sugar content from the body and honeydew of cotton aphid was observed, and inhibition of glycolysis and the TCA cycle resulted, which led to growth retardation in the cotton aphid. Based on plant-mediated RNAi in pest control offers high specificity, enabling precise targeting of pest genes while minimizing the impacts on non-target organisms. It has a durable effect, effectively suppressing the expression of pest genes. The process involves the Dicer enzyme randomly cleaving dsRNA, making it difficult for pests to develop resistance. Thus, these findings not only laid the foundation for understanding the relationship between the developmental retardation and inhibition of the glycolysis and TCA cycles of the cotton aphid treated with TG cotton but also provided a green and efficient pest control strategy to decrease honeydew contamination from cotton aphid and improve cotton quality based on TG cotton.

## 4. Materials and Methods

### 4.1. Insects and Cotton Plants

The susceptible cotton aphid population was collected in 2010 from Anningqu Town in Urumqi, Xinjiang, China and was fed in the Xinjiang Key Laboratory of Biological Resources and Genetic Engineering of Xinjiang University under insecticide-free conditions. The cotton aphid were reared on cotton seedlings (*G. hirsutum*) in a controlled insectarium at 25 ± 1 °C with a 50–60% relative humidity (RH) and a photoperiod of 14 h:10 h (light:dark). The transgenic cotton lines expressing ds*AgCYP6CY3-P1* (TG cotton lines) were obtained from our previous research [22].

### 4.2. RNA Sequencing (RNA-Seq) Data Analysis

The transcriptome data of cotton aphid feeding on non-transgenic cotton (NT cotton) and TG cotton for 48 h were obtained in the early stage of the project. A differential expression analysis of the two groups was performed using BMKCloud (Beijing Biomarker Technologies, Beijing, China) [56]. The significant differential expression genes (DEGs) were defined as *p* < 0.05 and |log2FoldChange| ≥ 1. The BMKCloud was applied for Kyoto encyclopedia of genes and genomes (KEGG) enrichment analysis, and the R language (https://www.r-project.org/, accessed on 22 March 2024) was used to plot the KEGG bubble plots. The KEGG pathway enrichment analyses were based on *p* < 0.05.

### 4.3. Extraction of Cotton Aphid Metabolites

A total of 1200 newborn nymphs were divided into six groups, with three groups fed on TG cotton and the remaining three groups fed on NT cotton. The cotton aphid was collected at 48 h from the treatment groups and control groups. Plastic cups were used to cover the cotton seedlings to prevent the cotton aphid from escaping. This was repeated three times. After 48 h, 200 cotton aphid were collected from each tube and sent to the China Huada Genome Bio-metabolism Company for testing. A total of 200 cotton aphid were weighed into a 2 mL EP tube with 800 µL of precooled extraction reagent (methanol:acetonitrile:water (2:2:1, *v*/*v*/*v*)), and then two magnetic beads were added, the sample was processed in a tissue grinder (50 Hz, 5 min) and the ground samples were stored at −20 °C for two hours. The samples were centrifuged at 15,000× *g* for 15 min at 4 °C. The supernatants were withdrawn and dried in a vacuum centrifuge and redissolved in 600 µL of 50% methanol. Next, the filtered samples were put in a sample vial for the LC-MS analysis [57].

### 4.4. UHPLC-MS/MS Analysis and Data Analysis

The separation and quantitative measurement of the metabolites were carried out using a tandem Q Exactive high resolution mass spectrometer (Thermo Fisher Scientific, Waltham, MA, USA, product code: 0725500) and a Waters UPLC I-Class Plus (Waters, Milford, MA, USA, product code: I-Class Plus). The chromatographic separation was performed on a Waters ACQUITY UPLC BEH C18 column (1.7 µm, 2.1 × 100 mm, Waters, USA), and the column temperature was maintained at 45 °C. The mobile phase consisted of 0.1% formic acid (A) and acetonitrile (B) in the positive mode, and in the negative mode, the mobile phase consisted of 10 mM ammonium formate (A) and acetonitrile (B). The gradient conditions were as follows: 0–1 min, 2% B; 1–9 min, 2–98% B; 9–12 min, 98% B; 12–12.1 min, 98% B to 2% B; and 12.1–15 min, 2% B. The flow rate was 0.35 mL/min, and the injection volume was 5 µL. Primary and secondary mass spectrometry data acquisition was performed using Q Exactive (Thermo Fisher Scientific, USA). The full scan range was 70–1050 *m*/*z* with a resolution of 70,000, and the automatic gain control (AGC) target for MS acquisitions was set to 3 × 10^6^ with a maximum ion injection time of 100 ms. The ESI parameters were set as follows: the sheath gas flow rate was 40, the aux gas flow rate was 10, the positive-ion mode spray voltage was 3.80, the negative-ion mode spray voltage was 3.20, the capillary temperature was 320 °C and the aux gas heater temperature was 350 °C. The raw data files generated by the UHPLC-MS/MS were processed using the Compound Discoverer 3.3 (CD 3.3, Thermo Fisher) to perform peak alignment, peak picking, and quantitation for each metabolite. A principal components analysis (PCA), partial least squares discriminant analysis (PLS-DA) and orthogonal partial least squares discriminant analysis (OPLS-DA) were performed at metaX (https://metax-tech.com/en, accessed on 5 March 2024) [58] (a flexible and comprehensive software for processing metabolomics data). Volcano plots were used to filter metabolites of sugar based on the log2 (FC) and log10 (*p*-value) of metabolites. For clustering heat maps, the data were plotted by a heatmap package in R language (https://www.r-project.org/, accessed on 5 March 2024). The functions of these metabolites and metabolic pathways were studied using the KEGG database. The metabolic pathways enrichment of differential metabolites was performed, and when the *p*-value of the metabolic pathway < 0.05, metabolic pathways were considered as statistically significant enrichment.

### 4.5. Quantitative Real-Time Polymerase Chain Reaction (RT-qPCR)

The RT-qPCR method was used to analyze the expression levels of glycolysis and the TCA cycle-related genes in the different samples. The reactions were carried out with a 20 µL mixture containing 10 µL of 2× TransStart Green qPCR SuperMix (TransGen Biotech, Beijing, China, code: AT341-02), 0.4 µL each of forward and reverse primers (10 µM each), 0.4 µL of an inert reference dye (50×) (optional), 7.8 µL of nuclease-free water, and 1 µL of cDNA was used as a template. The cycling conditions were as follows: 95 °C for 5 min followed by 40 cycles of 95 °C for 10 s and 56 °C for 30 s. The results were summarized as follows: The 18sRNA gene (GenBank Registry Number: DQ116441.1) was used as an internal control for the data normalization. The experiment was conducted in three independent biological replicates. The primers used in this study are shown in Table 1.

### 4.6. Determination of Enzyme Activities and Pyruvate Content in Glycometabolism

A total of 600 newborn nymphs were divided into six groups, with three groups fed on TG cotton and the remaining three groups fed on NT cotton. Cotton aphid were collected from the treated and control groups after 48 h for the determination of enzyme activity and pyruvate content. The 72 h samples were collected as above. Pyruvate carboxylase (PC), citrate synthase (CS), the malate dehydrogenase (MDH) activity and the pyruvate content of the cotton aphid were determined using the kit (Solarbio Co., Ltd., Beijing, China, Pyruvate Carboxylase Activity Assay Kit: BC0730, Citrate Synthase Activity Assay Kit: BC1060, NADP-Malate Dehydrogenase Activity Assay Kit: BC1050, Pyruvate Content Assay Kit: BC2200). Next, 20 mg of tissue was added to 200 µL of extraction solution and then homogenized at 0 °C. Subsequently, the homogenate was centrifuged at 8000× *g* for 10 min at 4 °C, and the supernatant was used for the determination of each enzyme activity and the pyruvate content. The absorbance was determined, and pyruvate carboxylase (PC), citrate synthase (CS), malate dehydrogenase (MDH) activity and pyruvate content were calculated based on their respective formulas [59].

### 4.7. Determination of Total Sugar Content

After 200 aphids were fed on NT and TG cotton for 36 h, 100 cotton aphid were collected from each plant for the determination of the total sugar content, tinfoil was placed underneath the cotton leaves, and the remaining 100 cotton aphid secreted honeydew for 24 h; then, honeydew was collected for the detection of the total sugar content of cotton aphid honeydew, which was determined by using the kit (Solarbio Co., Ltd., Beijing, China, Total Carbohydrate Content Assay Kit: BC2710). The 48 h, 72 h and 96 h periods were the same as the above mentioned three time points. The cotton aphid were homogenized with 1 mL of extraction solution or rinsed on aluminum foil and placed in a 95 °C water bath for 10 min. After cooling, the mixture was centrifuged at 4000× *g* for 10 min, the supernatant was used to measure the total sugar content, the absorbance was measured at the wavelengths specified by the kit and calculated, and the total sugar content was calculated according to the standard curve drawn.

### 4.8. Statistical Analysis

Each experiment was repeated three times independently, and the values were expressed as the mean ± standard deviation of the three independent experiments. All of the statistical analyses were performed using SPSS 20.0, and Graph Pad Prism 9.0 was used for graphing. The relative expression level was calculated using the 2^−ΔΔCT^ method [60]. The differences between the treatment groups and the control groups were statistically analyzed by an independent sample Student’s *t*-test (* *p* ≤ 0.05; ** *p* ≤ 0.01; *** *p* ≤ 0.001; **** *p* ≤ 0.0001).

## Figures and Tables

**Table 1:** Primer sequences.

Primer Name	Sequences (5′−3′)	Purpose
MIPP gene F	AAATCTCAAACACAACTCGGG	qRT-PCR
MIPP gene R	ATGGCATTCGTAAAGTACGGC	qRT-PCR
PC gene F	CTGATGTAGTTGATGTTGCTGTTG	qRT-PCR
PC gene R	TTTCCTGATTTCATTGTTGTTGTA	qRT-PCR
CS gene F	TCACATTATGCGATACCAAGC	qRT-PCR
CS gene R	CAAGTTCCAATACACCAAGCC	qRT-PCR
MDH gene F	CCTCTTCATCCGGCATCCAACA	qRT-PCR
MDH gene R	CAAACACCTTCCAGAACACCCA	qRT-PCR
18S rRNA F	CCGGAAAGATTGACAGATTGAG	qRT-PCR
18S rRNA R	CAGGACAGAGTCTCGTTCGTTATC	qRT-PCR

## Data Availability

The structural coordinates for the generated models will be provided upon reasonable request by email. The raw data supporting the conclusions of this article will be made available by the authors on request.

## Supplementary Materials

The supporting information can be downloaded at: https://www.mdpi.com/article/10.3390/ijms26010264/s1.
